# Protective Effect of Pyxinol, One Active Ingredient of *Lichenes* on Cisplatin-Induced Nephrotoxicity via Ameliorating DNA Damage Response

**DOI:** 10.3389/fphar.2021.735731

**Published:** 2021-09-06

**Authors:** Yanting Yang, Xiuhong Zhu, Guohua Yu, Jinbo Ma

**Affiliations:** ^1^ Department of Clinical Medicine, Binzhou Medical University, Yantai, China; ^2^ People’s Hospital of Jimo District, Qingdao, China; ^3^ Department of Pathology, Affiliated Yantai Yuhuangding Hospital, Medical College of Qingdao University, Yantai, China

**Keywords:** cisplatin, nephrotoxicity, pyxino, DNA damage response, p53, apoptosis

## Abstract

**Background:** Cisplatin is a valuable chemotherapeutic agent against malignant tumors. However, the clinical use of cisplatin is limited by its side effects such as renal injury. Pyxinol is an active constituent of *Lichenes* and its effects on cisplatin-induced nephrotoxicity is currently unknown. This study aims to examine the potential protective effects of pyxinol on cisplatin-induced renal injury and explore the underlying mechanisms.

**Methods:***In vivo* rat model of cisplatin-induced nephrotoxicity was induced by intraperitoneal (i.p) administration of cisplatin. The blood urea nitrogen and creatinine levels were measured and renal histological analysis was conducted to evaluate the renal function; The TUNEL staining, western blotting and real-time PCR assays were conducted to examine related molecular changes. Finally, the *in vivo* anti-tumor efficacy was examined in the xenograft tumor model using nude mice.

**Results:** Pretreatment with pyxinol attenuated cisplatin-induced increase in blood urea nitrogen, creatinine and urinary protein excretion and the magnitude of injury in the renal tubules. Pyxinol ameliorated the activation of p53 *via* attenuating the DNA damage response, which then attenuated the tubular cell apoptosis. Finally, pyxinol could potentiate the *in vivo* anti-tumor efficacy of cisplatin against the xenograft tumor of cervical cancer cells in nude mice.

**Conclusions:** Combining pyxinol with cisplatin could alleviate cisplatin-induced renal injury without decreasing its therapeutic efficacy, which might represent a beneficial adjunct therapy for cisplatin-based chemotherapeutic regimens in the clinic.

## Background

Cisplatin (cis-diamminedichloroplatinum II) is one of the most efficacious chemotherapeutic agents to treat solid tumors, such as non-small cell lung cancer, cervical cancer, testicular cancer (Pabla and Dong, 2008; Wang and Lippard, 2005) However, its use comes with several significant side effects. One of the major side effects of cisplatin use is nephrotoxicity, which is dose-dependent and as such limits its clinical use in certain patients (Arany and Safirstein, 2003; Pabla and Dong, 2008) Although current standard preventive protocols such as the hydration or diuretic therapies could decrease cisplatin-induced renal injury in many patients, a certain percentage of patients still suffer from cisplatin-induced nephrotoxicity (Perazella and Moeckel, 2010). Development of an effective adjunct therapy to curb or reduce cisplatin-induced nephrotoxicity could be a clinically beneficial approach to maximize its anti-tumor utility while reducing its side effects.

Despite decades of research attempting to uncover the molecular mechanisms attributable to cisplatin-induced nephrotoxicity, the exact mechanisms remain elusive. One of the increasingly recognized mechanisms underlying cisplatin-induced acute renal injury is DNA damage response (Yan et al., 2016; Zhu et al., 2015). Cisplatin is converted into a highly reactive molecule following aquation, which could bind to DNA and form intra-strand and inter-strand cross-linking. The cross-linking impedes DNA replication and/or transcription, causing DNA damage and ultimate cell apoptosis (Karasawa and Steyger, 2015; Zhu et al., 2015). As the guardian of genome, P53 would be immediately activated when the DNA damage occurred, to initiate the repair and/or the suicide program *via* regulating its downstream targets, such as *puma, bax*, *p21* (Jiang et al., 2004; Jiang et al., 2006; Man; Wei et al., 2007). Therefore, compounds that can attenuate the apoptosis *via* reducing DNA damage response could be potentially useful for the prevention and treatment of cisplatin-induced nephrotoxicity. Indeed, compounds such as lovastatin (Kruger et al., 2016) have been reported to demonstrate protective effect against cisplatin-induced nephrotoxicity *via* decreasing DNA damage response and attenuating tubular apoptosis.

**FIGURE 1:**
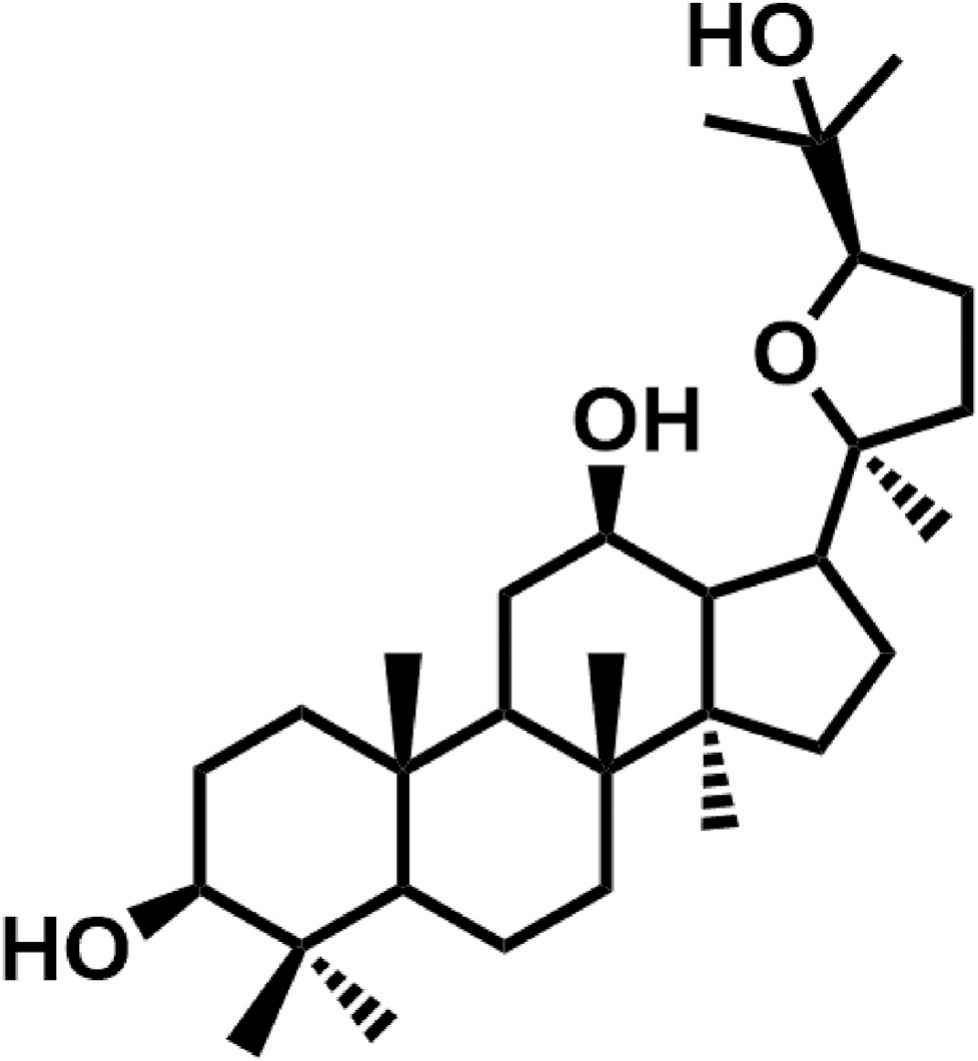
Chemical structure of pyxinol.

Natural products provide rich resources for discovery of drug candidates or leading compounds (Pan and Pan, 2010). Indeed, natural products-derived bioactive ingredients have been tested against common side effects of chemotherapeutic agents, such as the cardiomyopathy of doxorubicin and the nephrotoxicity of cisplatin, and several compounds with novel structures have been identified using well-validated models. For example, F11 (Wang et al., 2014) and Rh2 (Wang et al., 2012), two novel pseudoginsenosides, are reportedly able to attenuate cisplatin-induced nephrotoxicity and doxorubicin-induced cardiotoxicity, respectively. Mahadev Rao reported that cystone, a constituent from Himalaya herbals, showed protective effects against cisplatin-induced renal toxicity *via* inhibiting the lipid peroxidation (Mahadev Rao, 1998). Recently, we find that pyxinol, an active constituent of *Lichenes*, demonstrates strong protective activity against myocardial ischemia-reperfusion injury *in vitro* and *in vivo* (Sahu et al., 2011). As part of our continuing effort to discover novel protective agents against cisplatin-induced renal injury, here we report that pyxinol (Figure 1) was able to attenuate cisplatin-induced nephrotoxicity without affecting its anti-tumor efficacy.

## Materials and Methods

### Drugs and Chemicals

Pyxinol was prepared from 20 (S)-protopanaxadiol (PPD) as previously described (Bi et al., 2011). Briefly, after deriving 3, 12-diacetyl PPD with DMAP and Ac2O in pyridine, it was epoxidized with meta-chloroperbenzoic acid (m-CPBA) in dichloromethane to give 3, 12-diacetyl pyxinols. Then, 3, 12-diacetyl pyxinols was degraded with KOH in methanol to afford pyxinol (35% in three steps).

For *in vivo* study, pyxinol was dissolved in 0.5% carboxymethycellulose sodium (CMCS), and cisplatin (Qilu Pharm, Jinan, China) was dissolved in 0.9% sodium chloride solutions at proposed doses.

### Animals

Male Sprague-Dawley rats and female nude mice were provided by the Institute of Laboratory Animal Science, Chinese Academy of Medical Sciences (Beijing, China). All the animals were raised in a the room with room temperature of 21–22°C and the relative humidity of 60–65% under 12/12 h light cycle. Animals had free access to standard rodent chow and water. All of the experimental protocols were approved by the Experimental Animal Research Committee of Yantai University and conducted strictly following the Animal [Scientific Procedures] Act 1986).

### *In vivo* Rat Model of Cisplatin-induced Nephrotoxicity

Animals were randomly assigned into four groups. Nephrotoxicity was induced by intraperitoneal (i.p) administration of cisplatin dissolved in normal saline at the dose of 6.0 mg/kg body weight according to published literature (Wang et al., 2014). Animals in control group were given daily oral gavage of vehicle (0.5% carboxymethycellulose sodium (CMCS)) for 5 days. Animals in the pyxinol-treated group was given daily oral gavage of pyxinol dissolved in 0.5% (CMCS) at a dose of 10 mg/kg for 5 days. Right before the first pyxinol dosing, animals were treated with cisplatin dissolved in saline at a dose of 6 mg/kg only once. Five days after cisplatin treatment, rats were put into metabolic cages, and the 24-h urine samples were collected. Animals were euthanized thereafter. The kidney was harvested, weighed and calculated as percentage of the body weight. Blood and kidney samples were collected for the following analyses.

### Biochemical Analysis

Plasma creatinine and blood urea nitrogen levels as well as 24-h urinary protein excretion were measured as indicators of renal function. Briefly, the blood was firstly collected in heparinized tubes, and the plasma was separated by centrifugation. Plasma creatinine and blood urea nitrogen levels were detected using commercial test kits following published protocol (Yang et al., 2018; Humanes et al., 2012). The protein concentration of the urine samples were analyzed using Pierce™ BCA protein assay kit (Thermo Fisher Scientific, United States).

### Histological Analysis

Kidneys were isolated and fixed in 4% paraformaldehyde solution in PBS and then embedded in paraffin. Thereafter, tissue sections were performed for hematoxylin and eosin (HE) staining, in which the histopathological examination was conducted by a pathologist who was blind to the treatments following our published 4- point scale (Wang et al., 2014; Domitrovic et al., 2013): 0 = no damage, 1 = 0–20%, 2 = 20%–50%, 3 = 50–70%, and 4 = more than 70%. The mean score was calculated by counting at least 10 different fields for each sample.

### Western Blot Assay

Kidney tissue was lysed using RAPI buffer, and the supernatants were used for western blot assay according to published literature (Yang et al., 2018). Briefly, total proteins (30–60 μg) were separated in a 10–12% SDS-polyacrylamide gel for different target proteins, and then transferred onto a PVDF membrane. After blocking with 5% BSA, the membranes were incubated with p53, p-p53 (Ser-15), p-γH2ax, p21 puma, and bax antibodies, followed by incubation with peroxidase-conjugated goat anti-mouse IgG antibody (1:2000) for 2 h. Bands were visualized using the ECL-plus western blotting detection system.

### Terminal Deoxynucleotidyl Transferase dUTP Nick End Labeling (TUNEL)

The tubular cell apoptosis was detected with TUNEL staining using an apoptosis detection kit (*in situ* apoptosis detection kit) according to published protocol (Wang et al., 2010; Wang et al., 2011). Briefly, tissue sections (4 μm) were dewaxed and hydrated, and then permeabilized. Thereafter, the samples were incubated with 100 μl of reaction mixture containing the labeling enzyme and the TMR green labeled-dUTP at 37°C for 1 h in the moisture box. After extensive washing, the tissues were counterstained with DAPI and observed under a confocal fluorescence microscope, and the ratio of positive cells (the number of green cells divided by the number of the blue cells) was calculated for 10 different fields for each sample.

### Real-time Polymerase Chain Reaction (RT-PCR)

Total RNA of the tissues was prepared using TRIzol (Invitrogen, United States) as previously described (Wang et al., 2011). After reverse transcription using a first-strand cDNA synthesis kit (Fermentas, United States), the cDNA was subjected to RT-PCR assays using the ABI 7500 RT-PCR System. Primer sequences were as follows:

*P21*, F: 5′-CTG​GTG​ATG​TCC​GAC​CTG​TTC-3′, R: 5′-CTG​CTC​AGT​GGC​GAA​GTC​AAA-3′; *Bax,* F: 5′-GAT​CAG​CTC​GGG​CAC​TTT​AG-3′, R: 5′-TGT​TTG​CTG​ATG​GCA​ACT​TC-3′; *Puma*, F: 5′-AGT​GCG​CCT​TCA​CTT​TGG-3′, R: 5′-CAG​GAG​GCT​AGT​GGT​CAG​GT-3′; ***β**-actin*, F: 5′-CCC​AGC​ACA​ATG​AAG​ATC​AA GATCAT-3′, R: 5′-ATC​TGC​TGG​AAG​GTG​GAC​A GCGA-3′.

### Xenograft Tumor Model

Female babc/nu mice were used to establish the xenograft tumor model of human cervical cancer (Hela) as previously reported (Lv et al., 2017, Carvalho Rodrigues et al., 2013). Briefly, the tumors were implanted at the dorsum after isolated from donor animals, which were established by injecting the HeLa cells. When the xenograft tumors reached an average volume of 100–300 mm^3^, the animals were randomly assigned into four groups (*n* = 5): *1*) CMC group; *2*) 10 mg/kg pyxinol group; *3*) 3 mg/kg cisplatin group; *4*) 10 mg/kg pyxinol plus 3 mg/kg cisplatin group. The tumors were measured twice a week, and the relative tumor volume was calculated by the following formula: relative volume= (width)^2^ plus length/2. At the end of the experiment, the animals were sacrificed, and xenograft tumors were weighed and used to calculate the percentage of inhibition.

### Data Analyses and Statistics

The results were presented as mean ± SD. Comparisons between more than two groups were performed by analysis of variance (one way ANOVA) followed by Student t test. *P* < 0.05 was considered statistically significant unless indicated otherwise.

## Results

### Pyxinol Attenuated Cisplatin-induced Renal Injury

**FIGURE 2:**
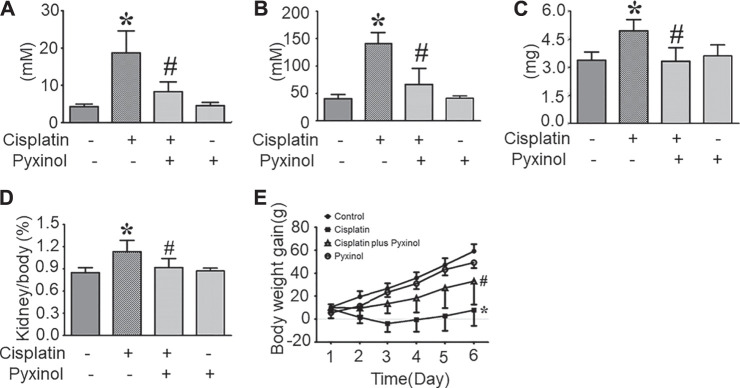
Effects of pyxinol on the levels of BUN and Cre in cisplatin-treated rats. **(A)** plasma BUN level; **(B)** plasma Cre level; **(C)** Total 24-h urinary protein; **(D)** Kidney body ratio; **(E)** Body weight gain. All data were expressed as means ± SD (*n* = 6). *p < 0.05, compared with the control group; *#p* < 0.05, compared with the cisplatin group.

Five days after cisplatin administration, the plasma BUN (blood urea nitrogen) and Cre levels, as well as 24-h urinary protein excretion, were significantly increased (Figure 2, *p* < 0.05, compared with the control group). 5 mg/kg pyxinol treatment significantly attenuated the increase of all the above biomarkers (*p* < 0.05, compared with the cisplatin group). Similar findings were observed on the body weight gain and the kidney/body ratio. Pyxinol alone showed no significant effect on any of the parameters.

### Pyxinol Ameliorated Cisplatin-induced Histopathological Changes

**FIGURE 3:**
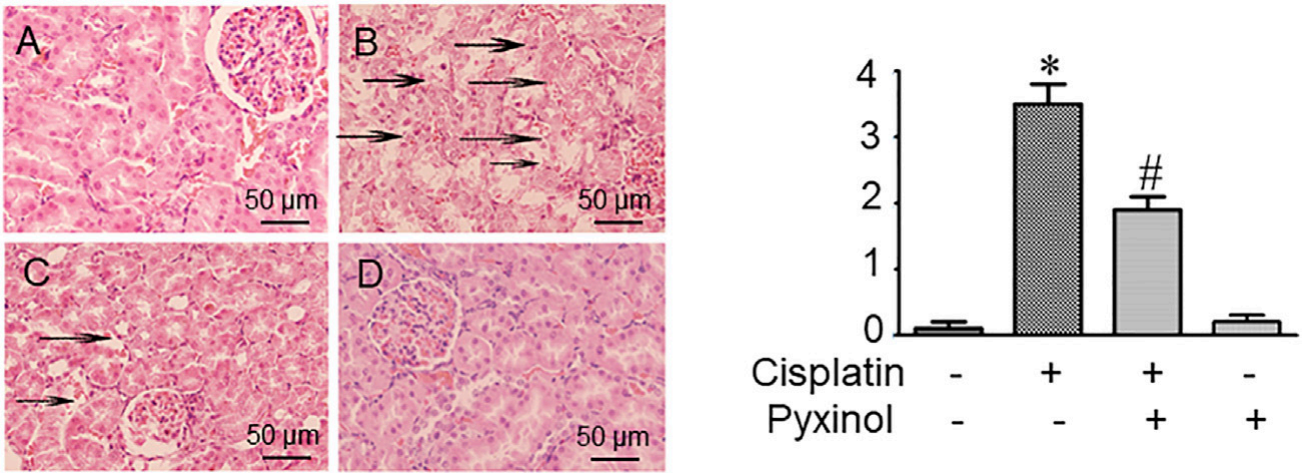
Effects of pyxinol on the kidney histopathological damage induced by cisplatin in rats. Left: **(A)** Control group; **(B)** Cisplatin group; **(C)** Cisplatin plus pyxinol group; **(D)** Pyxinol group. The damage levels of the renal tubules were blindly assessed based on a 4- point scale, and the scores were expressed as means ± SD (*n* = 6). Representative histopathological changes were indicated using the black arrows: cell necrosis, cell swelling and vacuolization (×400 magnifications). Right: quantification of the histological damage. *p < 0.05, compared with the control group; *#p* < 0.05, compared with the cisplatin group.

In histopathological examination, typical acute structural damages in the renal tubules, such as cell necrosis, cell swelling and vacuolization, and desquamation of epithelial cells, were observed in cisplatin-treated rats (Figure 3B). The histological injury score was significantly higher as compared to control animals (*p* < 0.05). Five days of pyxinol treatment significantly alleviated the histopathological injury (Figure 3C compared to Figure 3B), along with reduced injury score (*p* < 0.05, compared with cisplatin group).

### Pyxinol Reduced Cisplatin-induced Cell Apoptosis

**FIGURE 4:**
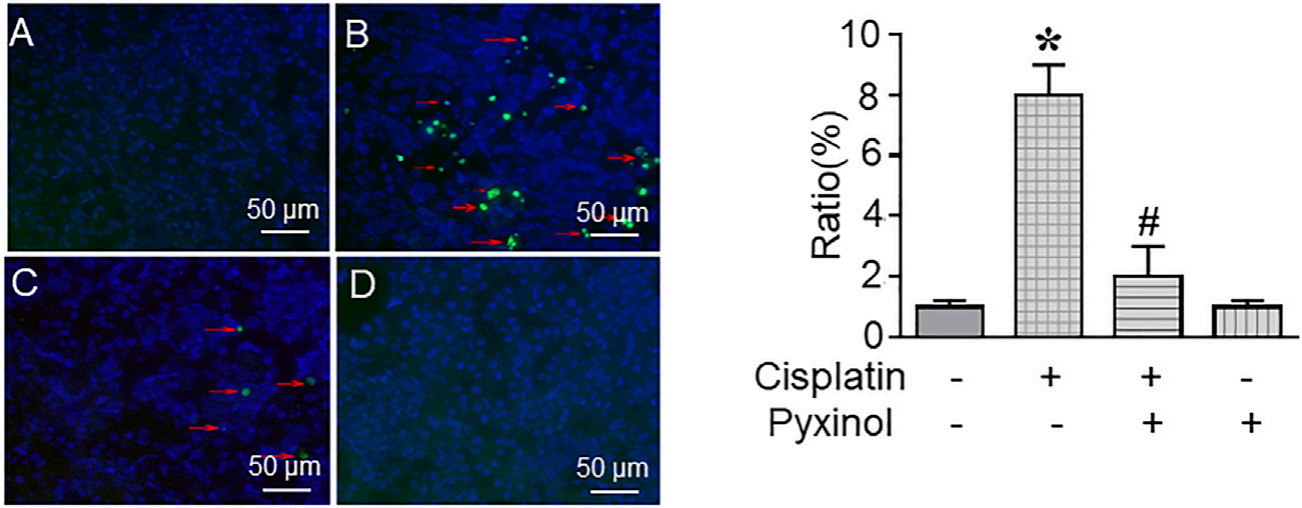
Effects of pyxinol on cisplatin-induced renal tubular cell apoptosis. The paraffin kidney section was stained with TUNEL staining, and the slides were observed using a confocal fluorescence microscope, in which at least 2,000 tubule cells (blue cells) were randomly examined to quantify the ratio of apoptotic cells (green cells). **(A)** Control group; **(B)** Cisplatin group; **(C)** Cisplatin plus pyxinol group; **(D)** Pyxinol group. The apoptotic cells were indicated with red arrows. *p < 0.05, compared with the control group; *#p* < 0.05, compared with the cisplatin group.

The ratio of TUNEL-positive cells in kidney tissue was significantly increased in cisplatin-treated rats as compared to control rats (Figure 4B, *p* < 0.05). Five days of pyxinol treatment significantly attenuated the ratio and the number of apoptotic tubule cells (Figure 4C, *p* < 0.05, compared with the cisplatin group).

### Pyxinol Attenuated Cisplatin-induced Increase of P53 Protein and its Downstream Molecules

**FIGURE 5:**
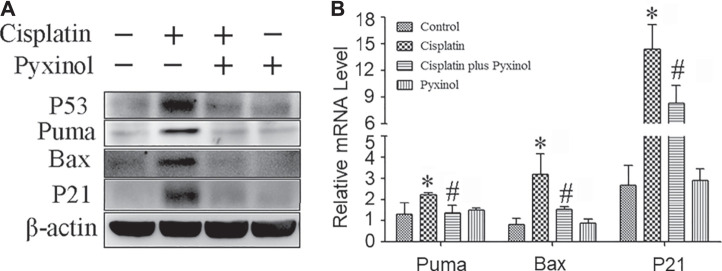
Effects of pyxinol on the expression of P53 and its transcription activity induced by cisplatin in rats. **(A)** Representative western blotting band; **(B)** quantification of mRNA levels as measured by real-time PCR. *p < 0.05, compared with the control group; *#p* < 0.05, compared with the cisplatin group.

To examine the potential molecular mechanisms, the protein and mRNA levels of p53 and its downstream molecules including p21, puma and bax were measured. Cisplatin treatment significantly increased the expression levels of both the proteins and mRNAs of the above signaling molecules (Figure 5), which were attenuated by pyxinol treatment. No significant changes were observed with these molecules in rats treated with pyxinol alone.

### Pyxinol Ameliorated Cisplatin-induced DNA Damage Response

**FIGURE 6:**
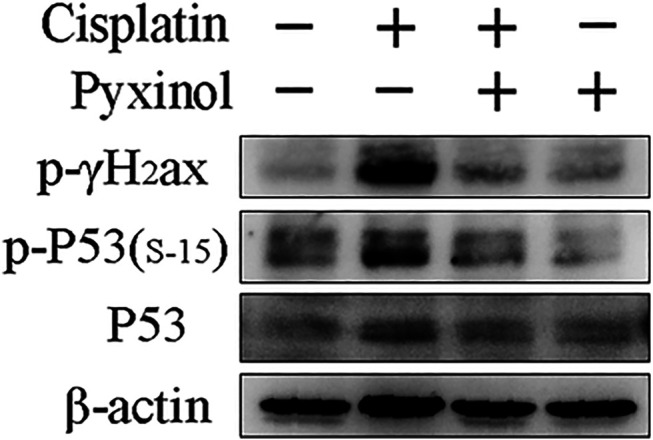
Effects of pyxinol on the phosphorylation of P53 and γH2ax induced by cisplatin in rats. Total protein expressions were detected using western blotting assay.

Cisplatin treatment increased the phosphorylation of p53 at serine 15, as well as the phosphorylation of γ-H2ax, two biomarkers of DNA damage (Figure 6). Pyxinol treatment attenuated the phosphorylation of p53 and γ-H2ax.

### Pyxinol did Not Attenuate the *in vivo* Anti-tumor Efficacy of Cisplatin

**FIGURE 7:**
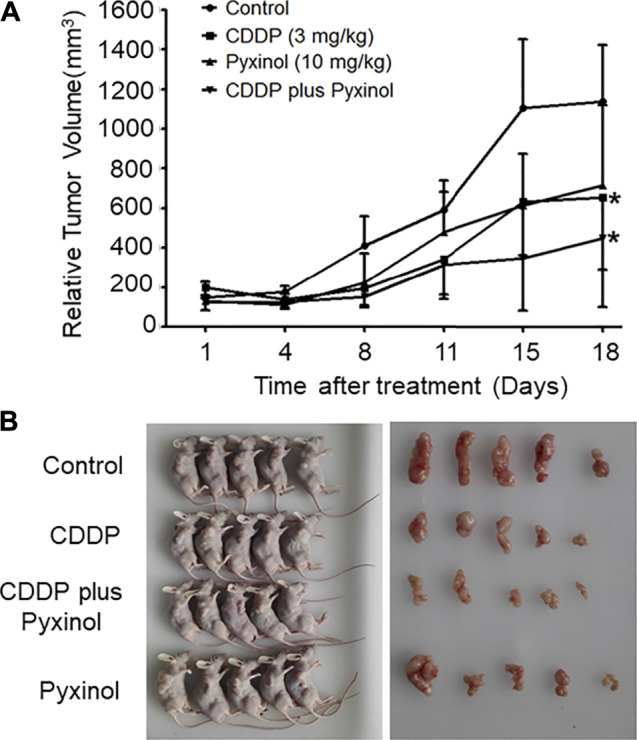
Effects of pyxinol on the *in vivo* anti-tumor activity of cisplatin in nude mice. The balb/c nu mice were transplanted with human cervical cancer xenograft tumors (Hela), and treated as indicated. After treatment, the relative tumor volume was measured. **(A)**: Tumor volume. All data are expressed as means ± SD (*n* = 5).*p < 0.05, compared with the control group. **(B)**: Photos of the mice and tumors.

To test the effect of pyxinol on the anti-tumor efficacy of cisplatin *in vivo*, the xenograft model in nude mice was established using human cervical cancer (Hela). At a dosage of 3 mg/kg, cisplatin significantly inhibited the growth of xenograft tumors (Figure 7, *p* < 0.05, compared with the control group; Table 1, *p* < 0.05, compared with the control group). Pyxinol (10 mg/kg) did not attenuate, but appeared to enhance, the anti-tumor activity of cisplatin (*p* = 0.38, compared with the cisplatin group). Pyxinol alone had no anti-tumor activity against cervical cancer *in vivo* (*p* = 0.66, compared with the control group).

**TABLE 1:** Effects of pyxinol on the antitumor activity of cisplatin in nude mice.

Group	Dosage (mg/kg)	Number	Body Weight (g)		Tumor Weight (g)	Inhibitor Rate (100%)
		Initial/End	Initial	End		
Control	0	5/5	21.8 ± 1.0	22.4 ± 1.4	0.81 ± 0.26	
Cisplatin	3	5/5	23.1 ± 1.1	22.0 ± 1.1	0.36 ± 0.17^a^	56.2
Pyxinol	10	5/5	23.5 ± 1.2	24.2 ± 1.2	0.47 ± 0.56	41.7
Cisplatin plus Pyxinol	3 plus 10	5/5	21.8 ± 1.2	21.6 ± 1.2	0.20 ± 0.13^a^	75.2

Data are expressed as means ± SD (*n* = 5).

a *p* < 0.05: compared with the control group.

## Discussion

Despite the well-established therapeutic efficacy against multiple solid tumors, side effects such as nephrotoxicity greatly limit the more extensive use of cisplatin in the clinic and available preventive strategies are only partially useful to reduce cisplatin-related toxicity. This reality inspires increasing research to develop adjunct therapies in the hope of finding combination therapy candidates with cisplatin to increase anti-tumor efficacy and/or reduce side effects (Arany and Safirstein, 2003; Perazella and Moeckel, 2010). In this study, we described the protective effects of a novel active constituent from *Lichenes*, pyixnol, on cisplatin-induced renal injury and examined the potential mechanisms underlying the apparent protective efficacy of pyixnol. We found that pyixnol demonstrated robust protective efficacy against cisplatin-induced renal injury as evidenced by drastically reduced BUN, creatinine and urinary protein excretion increase and the magnitude of renal tubule damage in cisplatin-treated animals. We also found that the protective effects of pyxinol were possibly achieved by attenuating the DNA damage response. Most importantly, these beneficial effects were achieved at the dose of pyxinol that did not affect the *in vivo* anti-tumor efficacy of cisplatin. Together, these results report for the first time the beneficial effects of pyxinol against cisplatin-induced nephrotoxicity and support the potential clinical utility of pyxinol as an adjunct to add to cisplatin chemotherapy.

It is well-established that the renal damage caused by cisplatin is at least partially due to the accumulation of cisplatin in the S3 segment of the renal tubules (Cristofori et al., 2007). As a result, the activity of the glomerular filtration is decreased and the concentration of plasma BUN and Cre is increased. Indeed, the animals in this study demonstrated significantly increased plasma BUN and CRE levels and content of albumen in the urine 5 days after a single injection of 6 mg/kg cisplatin, suggesting glomerulus damage. Pyxinol treatment significantly ameliorated all the above biomarkers. Similar findings were observed in the histopathological examinations, in which the representative changes such as cell necrosis, the desquamation of epithelial cells around proximal tubule, were all greatly improved after pyxinol treatment. These results provide strong evidence for the protective efficacy of pyxinol in cisplatin-induce renal injury.

One underlying mechanism of cisplatin-induced nephrotoxicity is cisplatin-provoked cell apoptosis, in which the oxidative stress and DNA damage-induced p53 activation acts as an initiator (Jiang et al., 2006; Yang et al., 2014). Consistent with the literature (Galgamuwa et al., 2016; Wang et al., 2014), we found that the renal tubule cells showed higher apoptosis ratio after treatment with cisplatin. The ratio of apoptotic cells was significantly decreased by pyxinol treatment, suggesting the protective effect of pyxinol against cisplatin-induced cell apoptosis in renal tubule. Interestingly, unlike our previous findings (Wang et al., 2014), pyxinol had no significant effect on cisplatin-induced oxidative stress in the kidney (data not shown). Similarly, pyxinol treatment also significantly attenuated cisplatin-evoked increase in the expression of p53 and its target genes such as p21, puma, bax as well as their protein levels, which are well-known players in the regulation of cisplatin-induced renal apoptosis (Yang et al., 2014). These data clearly showed that pyxinol could ameliorate the expression of p53 and suppressed its transcription after cisplatin exposure in the kidney.

There is clear evidence that DNA damage response is a crucial mechanism contributing to cisplatin-induced acute kidney injury. Following aquation, cisplatin could bind to DNA and form cross-linking in the kidney tubular cells, which causes replication stress and DNA damage (Wang and Lippard, 2005; Karasawa Steyger, 2015; Zhu et al., 2015). As the “sensors” of DNA lesions, ATM and ATR kinases phosphorylate p53 and γ-H2AX protein, two important biomarkers for the DNA damage, and initiate DNA repair, or induce cell cycle arrest and cell apoptosis. As expected, we found that cisplatin induced the phosphorylation of P53 (Ser-15) and γ-H2AX in the kidney tissue and pyxinol treatment attenuated such increases. These results suggest that pyxinol suppressed p53 activation was at least partially mediated *via* the decrease of cisplatin-induced DNA damage response.

**FIGURE 8:**
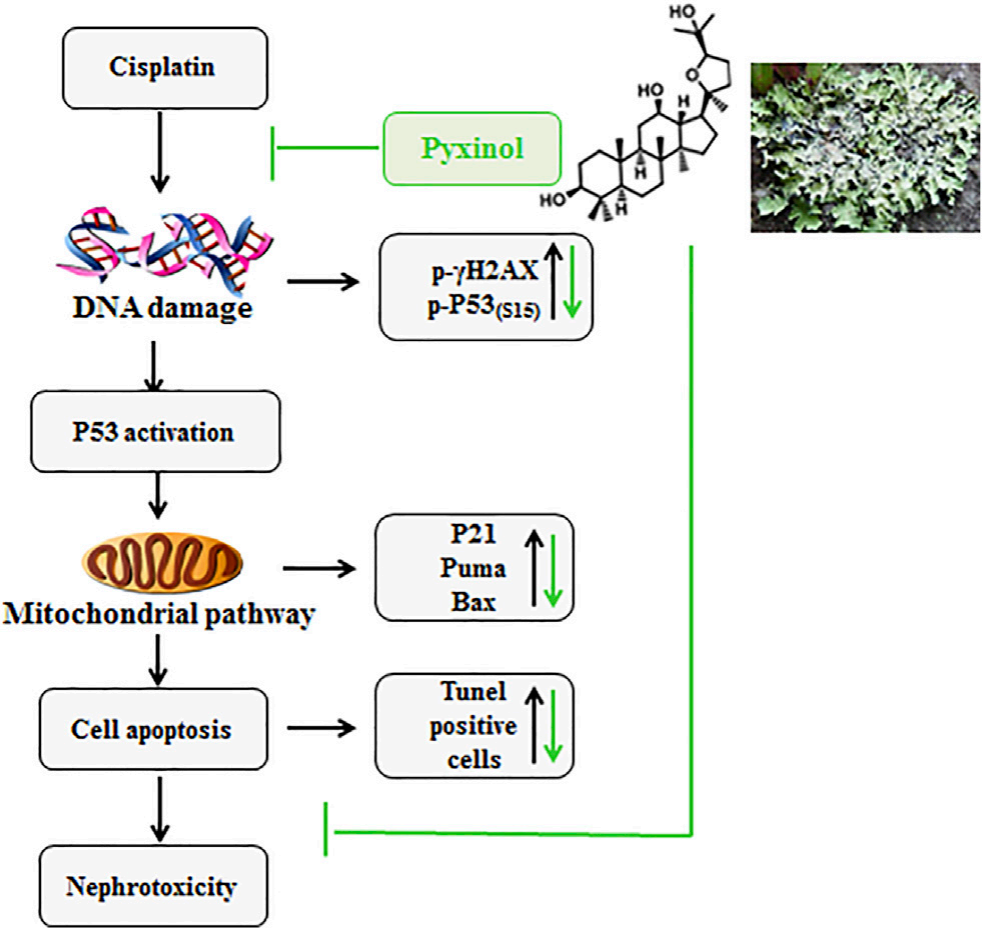
The working model.

As a potential adjunct therapy candidate, one crucial prerequisite is that pyixnol should not affect the anti-tumor efficacy of cisplatin. This possibility was tested using the xenograft tumor model in nude mice, and the results clearly showed that pyxinol did not affect the anti-tumor activity of cisplatin *in vivo*. A schematic model was proposed (Figure 8) to show the process and mechanism how pyixnol protects against cisplatin-induced renal injury.

## Conclusion

Our study showed for the first time that pyxinol, one active constituents of *Lichenes*, was protective against cisplatin-induced nephrotoxicity through inhibiting tubular cell apoptosis *via* deceasing the DNA damage response. Importantly, pyxinol did not attenuate the anti-tumor efficacy of cisplatin, and therefore might be a novel potential adjunct therapy for cisplatin-based chemotherapeutic regimen in the clinic.

## Data Availability

The data for the current study are available from the corresponding author on reasonable request.

## Abbreviations

BUN, blood urea nitrogen; CMCS, carboxymethycellulose sodium; Cre, creatinine; HE, hematoxylin and eosin; i.p, intraperitoneal; m-CPBA, meta-chloroperbenzoic acid; PPD, 20 (S)-protopanaxadiol; RT-PCR, real-time polymerase chain reaction; TUNEL, terminal deoxynucleotidyl transferase-mediated dUTP nick end-labeling.
