# Supplementary figures and images for: Protective Effect of Pyxinol, One Active Ingredient of Lichenes on Cisplatin-Induced Nephrotoxicity via Ameliorating DNA Damage Response
Source: Front Pharmacol. 2021 Sep 6;12:735731. doi: 10.3389/fphar.2021.735731 (PMC8450395; doi:10.3389/fphar.2021.735731)

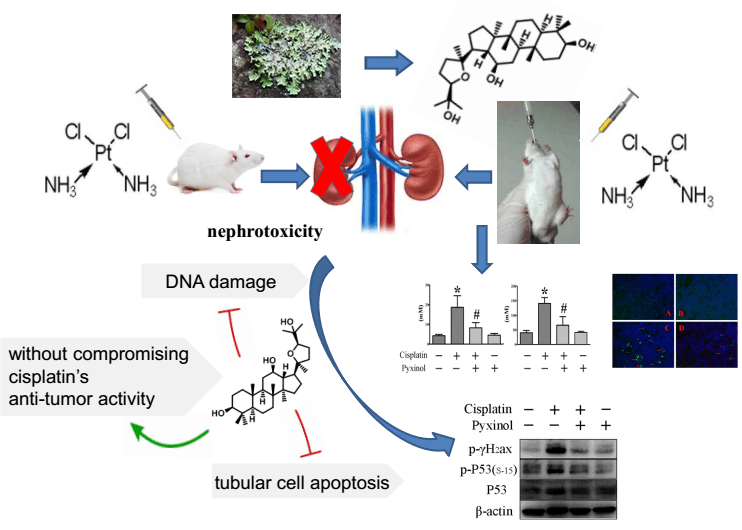

Supplement: Supplementary file 1 [file Image1.TIF]
